# Functional Characterisation of the Circular RNA, *circHTT(2-6)*, in Huntington’s Disease

**DOI:** 10.3390/cells12091337

**Published:** 2023-05-07

**Authors:** Laura Gantley, Brett W. Stringer, Vanessa M. Conn, Youichirou Ootsuka, Duncan Holds, Mark Slee, Kamelya Aliakbari, Kirsty Kirk, Rebecca J. Ormsby, Stuart T. Webb, Adrienne Hanson, He Lin, Luke A. Selth, Simon J. Conn

**Affiliations:** 1 Flinders Health and Medical Research Institute (FHMRI), College of Medicine and Public Health, Flinders University, Bedford Park, SA 5042, Australia; laura.gantley@flinders.edu.au (L.G.);; 2 Lifelong Health, South Australian Health and Medical Research Institute, Adelaide, SA 5000, Australia; 3 Centre for Neuroscience, Human Physiology, College of Medicine and Public Health, Flinders University, Bedford Park, SA 5042, Australia; 4 Department of Genetics and Molecular Pathology, SA Pathology, Flinders Medical Centre, Bedford Park, SA 5042, Australia; 5 Freemasons Centre for Male Health and Wellbeing, Flinders University, Bedford Park, SA 5042, Australia; 6 Adelaide Medical School, University of Adelaide, Adelaide, SA 5000, Australia

**Keywords:** circular RNA, Huntington’s disease, *Huntingtin*, *HTT*, triplet repeat disorders

## Abstract

Trinucleotide repeat disorders comprise ~20 severe, inherited, human neuromuscular and neurodegenerative disorders, which result from an abnormal expansion of repetitive sequences in the DNA. The most common of these, Huntington’s disease (HD), results from expansion of the CAG repeat region in exon 1 of the *HTT* gene via an unknown mechanism. Since non-coding RNAs have been implicated in the initiation and progression of many diseases, herein we focused on a circular RNA (circRNA) molecule arising from non-canonical splicing (backsplicing) of *HTT* pre-mRNA. The most abundant circRNA from *HTT*, *circHTT(2-6)*, was found to be more highly expressed in the frontal cortex of HD patients, compared with healthy controls, and positively correlated with CAG repeat tract length. Furthermore, the mouse orthologue (mmu_*circHTT(2-6)*) was found to be enriched within the brain and specifically the striatum, a region enriched for medium spiny neurons that are preferentially lost in HD. Transgenic overexpression of *circHTT(2-6)* in two human cell lines—SH-SY5Y and HEK293—reduced cell proliferation and nuclear size without affecting cell cycle progression or cellular size, or altering the CAG repeat region length within *HTT*. *CircHTT(2-6)* overexpression did not alter total HTT protein levels, but reduced its nuclear localisation. As these phenotypic and genotypic changes resemble those observed in HD patients, our results suggest that *circHTT(2-6)* may play a functional role in the pathophysiology of this disease.

## 1. Introduction

Huntington’s disease (HD) is a rare, largely inherited, autosomal disease that affects 1–5 people in 100,000 [1]. HD is the most common trinucleotide repeat disorder, resulting from the expansion of the CAG repeat region in exon 1 of the *Huntingtin* (*HTT*) gene [2]. This results in the formation of an extended polyglutamine tract generating a mutant HTT (mHTT) protein, causing misfolding, aggregation, and an increased capacity for non-canonical protein interactions [3]. The cause(s) of the expansion of the CAG repeat regions and the function of the altered HTT protein remain highly debated. However, a significant body of work has implicated HTT in a range of biological processes, such as axonal trafficking, gene regulation, impairment of the ubiquitin-proteasome system, and cell autophagy [3]. Furthermore, *HTT* knockout animal models are embryonic lethal, highlighting the essential function(s) of the HTT protein [4,5].

While healthy individuals can have expansion of the CAG repeat region, HD pathogenesis is observed at or above a threshold of 36 repeats [3]. The CAG repeat region has been reported to expand over time, with 4% of HD cases considered sporadic, as the individual’s parents both lack pathogenic repeat lengths [1]. A higher number of repeats is associated with an earlier disease onset and increased severity of symptoms [6]. The neurodegenerative effect of HD is believed to result from a loss of medium spiny neurons (MSN) within the striatum of HD patients, causing overactivation of the thalamus [7]. It is this overactivation that results in the most characteristic symptom of HD, chorea, which is uncontrollable movement of the body [6]. HD patients will also suffer from cognitive and psychiatric symptoms, with the peak onset of symptoms delayed until ~40–50 years of age.

The roles of alternative splicing (AS) and noncoding RNAs have been of great interest for neurological diseases, due to their well-established enrichment in neurological tissue [8]. RNA splicing converts precursor mRNA into mature mRNA through the removal of introns and sequential ligation of canonical exons. However, it is estimated that 90–95% of all eukaryotic genes are alternatively spliced (AS), with these non-canonical RNA transcripts representing the greatest source of expansion of the functional transcriptome and proteome [8,9]. AS is a dynamic, well-orchestrated, and cell-type specific process which plays critical roles in cell differentiation and the generation of highly tissue-specific proteins, particularly in the brain, which has the highest rate of AS in the human body [10]. Perhaps unsurprisingly, misregulation of alternative splicing through mutations of RNA splicing factors and RNA splice sites can be devastating, with evidence of aberrant splicing found in cancer and neurological diseases [11].

**Figure 1:**
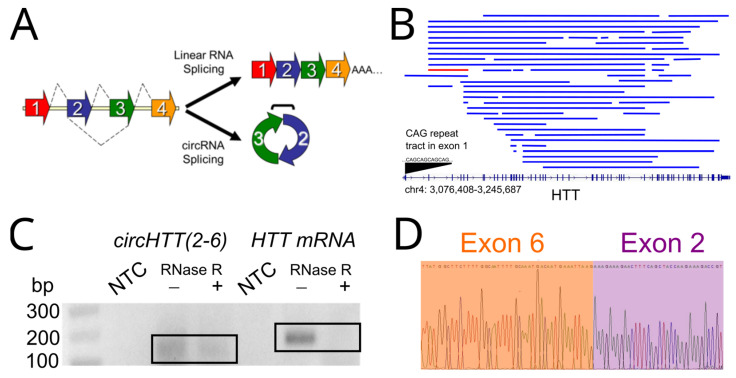
*CircHTT(2-6)* is the predominant circRNA expressed from the *HTT* gene. (**A**) Linear vs. circular RNA splicing, with exons shown as coloured arrows. (**B**) All known circRNAs from the *HTT* gene from circBase and CIRCpedia v2. *HTT* gene locus shown at bottom, with vertical blue lines representing exons. Blue lines represent individual circRNAs that arise from the HTT gene, with *circHTT(2-6)* highlighted in red. CAG repeat region in exon 1 is highlighted. (**C**). Agarose gel electrophoresis showing PCR amplicons for *circHTT(2-6)* and *HTT* mRNA from SH-SY5Y cells with and without RNase R treatment prior to cDNA synthesis. NTC: non-template control. (**D**) Sanger sequencing chromatogram of *circHTT(2-6)* amplicon showing backsplice junction between exons 6 and 2.

The most recently identified class of alternatively-spliced RNAs, circular RNAs (circRNAs) are covalently closed, single-stranded, and largely non-coding RNA transcripts [12]. High-throughput RNA sequencing, and improvements in computational identification of noncoding RNAs, has yielded a growing database of over 200,000 circRNAs within the human transcriptome, which primarily consist of exonic sequences [13]. CircRNAs are formed co-transcriptionally, and their biogenesis is in competition with canonical linear RNA splicing (Figure 1A) [14]. CircRNA biogenesis is regulated primarily by two non-mutually exclusive mechanisms: (1) the presence of inverted, complementary repeats in flanking introns; and (2) interaction with RNA binding proteins to promote (QKI, FUS, MBNL, NOVA1, NOVA2) or inhibit (ADAR1, mediating adenine to inosine editing) circularisation [13,15].

Differential expression of circRNAs has been identified in another neurological disease, Alzheimer’s disease (AD). CircRNA ciRS-7 was not only downregulated in AD patients, but also drove downregulation of UPLA, which is vital for the clearance of amyloid peptides, which accumulate in AD [16]. The presence and potential roles of circRNAs in HD have yet to be explored.

Within this study, we determined that a circRNA from the *HTT* gene comprising exons 2, 3, 4, 5, and 6—*circHTT(2-6)*—is more abundant within HD patient brains compared to healthy controls, and is strongly positively correlated with CAG repeat length. Overexpression of *circHTT(2-6)* in two human cell lines (SH-SY5Y and HEK293 cells) resulted in phenotypic effects consistent with HD. Thus, we hypothesise that *circHTT(2-6)*, the first characterised circRNA from the *HTT* gene, may have roles in the progression of HD.

## 2. Materials and Methods

### 2.1. Human Ethics Approval and Patient Tissue Samples

For all primary tissue sourced for these experiments, written informed consent was obtained from each subject or from their guardian. Specimens were received from the South Australian Brain Bank (SABB, Adelaide, Australia) and included samples from seven patients with a clinical diagnosis of HD and four samples from patients with no neurological manifestations (hereafter referred to as healthy, or control tissue; Table 1). Ethical approval for this project was provided by the Southern Adelaide Local Health Network Human Research Ethics Committee (SALHN HREC, Adelaide, Australia), project approval number 5198. The SABB is approved by the Southern Adelaide Clinical Human Research Ethics Committee (SAC HREC, Adelaide, Australia), approval number 51.045. Post-mortem fresh brain was sectioned in approximately 10 mm coronal slices, numbered from frontal to occipital regions, snap-frozen, and stored at −80 °C. Cores (6 mm diameter) of cerebral cortex within the posterior frontal lobe were obtained from the frozen brain section by laying them on a bed of dry ice and using a sterilised metal corer. Cores were subsequently placed into pre-chilled, sterile cryovials and immediately snap-frozen in dry ice to preserve RNA integrity and histology. Diagnostic PCR for CAG trinucleotide repeat expansion in exon 1 of *HTT* of patient material was performed by SA Pathology diagnostics, with both alleles reported herein.

### 2.2. Mouse Major Organ and Brain Tissue Harvesting

Tissues were harvested by dissection from four female C57/BL6 mice and included nine major organs (brain, liver, spleen, thymus, ovary, kidney, skin, lung, and heart) and eight brain regions (medulla, pons, midbrain, olfactory bulb, cerebellum, striatum, hippocampus, and hypothalamus). This material was immediately placed into RNALater^®^ (ThermoFisher Scientific, Waltham, MA, USA) after microdissection and stored at −20 °C until use. Tissue collection was approved by the Flinders University Animal Welfare Committee (animal ethics approval number AERP6001-1).

### 2.3. RNA and DNA Purification from Tissue

Human and mouse tissue samples (approximately ~20–100 mg) were transferred to a BeadBug™ 2 mL tube prefilled with triple-pure, high impact 3.0 mm Zirconium beads (Z763802; Merck, Darmstadt, Germany) on dry ice, and 1 mL TRIzol™ (ThermoFisher Scientific, Waltham, MA, USA) was added. Samples were homogenised at 4 °C using the PreCellys Evolution (Bertin Instruments, Rockville, MD, USA) for 3 × 10 s cycles at 6500 rpm, with 30 s pauses between cycles. RNA was subsequently purified and DNase-treated using the RNEasy^®^ Lipid Tissue mini kit (Qiagen, Venlo, The Netherlands) according to the manufacturer’s instructions. RNA was quantified using the N80 nanophotometer (Implen, Munich, Germany) and RNA quality assessed by LabChip GX (Perkin Elmer, Waltham, MA, USA). 

### 2.4. Cell Culture

HEK293 cells were cultivated at 37 °C with 5% CO_2_ in air in DMEM (Merck, Darmstadt, Germany) supplemented with 10% *v*/*v* foetal bovine serum (Bovogen, East Keilor, Australia) and 1 mg/mL Antibiotic-Antimycotic (Merck, Darmstadt, Germany). SH-SY5Y cells were cultivated at 37 °C with 5% CO_2_ in air in DMEM (Merck, Darmstadt, Germany) supplemented with 10% *v*/*v* heat-inactivated foetal bovine serum (Bovogen, East Keilor, Australia). Cells were serially passaged prior to reaching 90% confluence using TrypLE express (ThermoFisher Scientific, Waltham, MA).

### 2.5. Generation of circRNA Overexpression Constructs

Overexpression of human *circHTT(2-6)* was performed by cloning sequentially 1000 bp from the distal end of intron 1 of the *HTT* gene, followed by exons 2–6 and the proximal 200 bp of intron 6 into pcDNA3.1-neo. An additional 800 nt of *HTT* intron 1 was added downstream of this, in reverse complement, in order to promote circularisation of the exonic sequence, as described previously [15,17]. Sanger sequencing was performed to confirm successful construction of the vector. Two human cell lines, HEK293 and SH-SY5Y, were transfected with empty vector (pcDNA3.1-neo) or overexpression vector (*circHTT(2-6)*) using Lipofectamine 2000 (ThermoFisher Scientific) in 6-well tissue culture plates (Sarstedt, Mawson Lakes, Australia) as recommended by the manufacturer. Stable, polyclonal cell populations were selected for seven days using 1300 μg/mL G418 for HEK293 and 350 μg/mL G418 for SH-SY5Y, in order to establish three biological replicates for each cell line. After seven days of selection, RNA was harvested from the transfected cell lines and RT-PCR was performed as described below. A single PCR product was produced using circRNA-specific primers and Sanger sequencing confirmed it contained the correct backsplice junction. QRT-PCR was also performed in order to quantify *circHTT(2-6)* expression levels compared to empty vector (EV) controls, using *TBP* or *GAPDH* as housekeeping genes as previously described [18].

### 2.6. RT-PCR/qRT-PCR

RNA was harvested from cell pellets (1–3 million cells) using 0.5–1 mL TRIzol™ (ThermoFisher Scientific) and purified with a Direct-zol™ RNA miniprep kit (Zymo Research, Irvine, CA, USA) with on-column DNase I treatment. RNA was quantified using a NanoDrop One Microvolume UV-Vis Spectrophotometer (ThermoFisher Scientific). RNase R treatment was performed on two micrograms of total RNA as previously reported [19]. Total RNA or RNase R-treated RNA was reverse transcribed with a QuantiTect^®^ reverse transcription kit (Qiagen). After RT, QuantiTect SYBR^®^ Green PCR Kit (Qiagen, USA) was used for qRT-PCR, normalising to *GAPDH* (mouse/human) and/or *TBP* (human). All oligonucleotides used in this study were ordered from Integrated DNA Technologies (Singapore, Singapore), with sequences provided (Supplementary Table S1).

### 2.7. Western Blotting

Total soluble protein was harvested from HEK293 and SH-SY5Y cells using RIPA buffer with 1× protease inhibitor (mini-EDTA free) and phosphatase inhibitor cocktails (ThermoFisher Scientific, USA). Protein was quantified by Bradford Assay (BioRad, Hercules, CA, USA), and 25 μg of protein was loaded on Any kD™ Mini-PROTEAN^®^ TGX Stain-Free™ precast PAGE gels (BioRad) and imaged using ChemiDoc imager (BioRad). Total protein loading was quantified and used to normalise between samples using the ChemiDoc software. Membranes were blocked with 5% skim milk powder in tris buffered saline with 0.1% (*v*/*v*) Tween-20 (TBS-T) for 1 h at room temperature. Anti-HTT, rabbit monoclonal primary antibody (ab109115; Abcam, Cambridge, UK) was diluted 1:1000 in blocking solution and probed overnight at 4 °C with rocking. Following five washes with TBS-T, goat anti-rabbit HRP-conjugated secondary antibody (ThermoFisher Scientific) at 1:10,000 dilution was used as a secondary antibody. After a further five washes, chemiluminescent detection was carried out using SuperSignal West Pico PLUS reagent (ThermoFisher Scientific). Precision Plus Protein™ Kaleidoscope™ Prestained Protein Standard (Bio-Rad) was used for size estimation.

### 2.8. Proliferation Assay

Cells were seeded into a 96-well plate (2000 cells/well) with six technical replicates of each cell line. Plates were incubated in the InCucyte SX5 (Sartorius, Göttingen, Germany), with each well imaged every 2 h for up to 72 h. Analysis was performed using Incucyte^®^ Software (v2020C) to identify cell boundaries, thus allowing for cell confluency to be calculated as phase confluence (%). Exponential growth phases were identified for each cell line as the 10 h period where cell growth increased at the fastest rate.

### 2.9. High-Content Cell Morphology Analysis

HEK293 or SH-SY5Y cells were seeded into a 384-Well Nunc™ black plate with optically clear polymer bottom (ThermoFisher, USA) at 1000 cells/well and with six replicates of each cell line. After 24 h, cells were washed with 1× PBS and then fixed with 4% paraformaldehyde for 10 min at room temperature. Cells were gently washed three times with 1X PBS. Cells were stained with 1 μM CellTracker™ Deep Red Dye (ThermoFisher, USA) and 1:1000 dilution of Phalloidin-iFluor 488 (Abcam) in 1X PBS and incubated at room temperature for 1 h. Cells were then stained with 1 µg/mL DAPI in 1X PBS for 10 min at room temperature, washed 3 times with 1X PBS, and then stored at 4 °C in 50 µL of PBS until analysis by the Operetta CLS Imaging System (Perkin Elmer, USA). Analysis was done with Harmony High-Content Imaging and Analysis Software ver4.8 (Perkin Elmer, USA).

### 2.10. Immunofluorescence

At 24 h prior to fixation, cells were seeded at 30% confluency on 13 mm round, glass coverslips in a 24-well plate. Cells were allowed to attach overnight, and then were fixed with 4% paraformaldehyde for 10 min at room temperature. Cells were then washed 3 times with ice-cold, 1X PBS and permeabilised with 0.1% Triton X-100 in 1X PBS for 10 min at room temperature. Cells were again washed with 1X PBS for 5 min, 3 times. Cells were then blocked with 1% (*w*/*v*) BSA and 22.52 mg/mL glycine in PBS-T for 30 min with gentle rocking. Cells were then incubated with primary antibody, anti-HTT RabMab^®^ Rabbit monoclonal antibody (Abcam, ab109115), at 1:1000 in PBS-T containing 1% BSA for 1 h at room temperature in the dark. Cells were then washed with PBS-T for 5 min, 3 times, and then incubated in the dark with the secondary antibody (goat anti-rabbit Alexa Fluor™ Plus 647; ThermoFisher Scientific, USA) at 1:10,000 dilution in PBS-T with 1% BSA for 1 h at room temperature. Cells were then washed 3 times with PBS-T for 5 min. Cells were incubated with DAPI at 1 µg/mL in 1X PBS for 10 min at room temperature in the dark. Cells were then washed with PBS for 5 min, 3 times. Glass coverslips were then mounted onto glass slides using 20 µL of buffered glycerol and sealed with clear nail polish. Slides were imaged using an Olympus AX70 microscope at 100× magnification with oil immersion lens. Quantification of fluorescence intensity (density) for nucleus, cytoplasm, and perinucleus, was performed using ImageJ according to [20] and presented as fluorescence density by dividing the total fluorescence signal by the total pixel area for each region.

### 2.11. Huntington’s Disease Diagnostic PCR

Genomic DNA was harvested with a GenElute™ Mammalian Genomic DNA Miniprep kit (Sigma-Aldrich). Samples were then submitted to SA Pathology to perform the Huntington’s Disease direct test PCR assay using reporting primers HD1 (6′FAM-labeled) and 447X primers (Supplementary Table S1). Samples were performed in duplicate using 100 ng of DNA. All PCR products were separated via capillary electrophoresis and imaged with the 3130xl Genetic Analyzer (ABI Prism) with fragment analysis using PeakScanner™ software ver1.0 (ThermoFisher, USA).

## 3. Results

### 3.1. CircRNAs from the Huntingtin (HTT) Gene

In addition to exhibiting cell-type-specific and tissue-specific expression, a number of circRNAs have been shown to be evolutionarily conserved, reinforcing their potential to play functional roles [12]. However, whether circRNAs can influence HD, the most common, monogenic triplet repeat expansion disorder, is unknown. In order to address this question, we searched 2 circRNA public databases, circBase (http://www.circbase.org, accessed on 2 March 2023) and CIRCpedia v2 (http://yang-laboratory.com/circpedia/search, accessed on 2 March 2023), to identify *HTT*-derived circRNAs, and identified 54 circRNAs (Figure 1B, Supplementary Table S2). The most highly expressed of these circRNAs across a variety of cell types, called hsa_circ_0001392 and HSA_CIRCpedia_48392, comprises exons 2–6, (chromosome 4: 3,074,408–3,247,687 from hg19 genome assembly), hereafter referred to as *circHTT(2-6)*. Furthermore, *circHTT(2-6)* is adjacent to the CAG repeat expansion site in exon 1 that is responsible for the mutant Huntingtin (mHTT) protein.

### 3.2. circHTT(2-6) Is a Bona Fide circRNA Enriched in HD Patient Brains

In order to confirm that *circHTT(2-6)* is a bona fide circular RNA, we harvested RNA from the neuroblastoma cell line, SH-SY5Y, which is commonly used as an in vitro cell model in HD and other neurodegenerative diseases [21,22]. This purified RNA was RNase R-treated or mock-treated and RT-PCR was performed with both *circHTT(2-6)*-specific and linear *HTT* primer pairs. RNase R can digest linear RNA, whilst leaving the circRNAs largely unaffected due to their lack of a 5′ or 3′ tail. A single amplicon of the expected size (129 bp) and exon composition was seen for both RNase R-treated and mock-treated samples (Figure 1C,D). In contrast, the cognate linear mRNA, *HTT*, was not able to be amplified following RNase R digestion.

**Figure 2:**
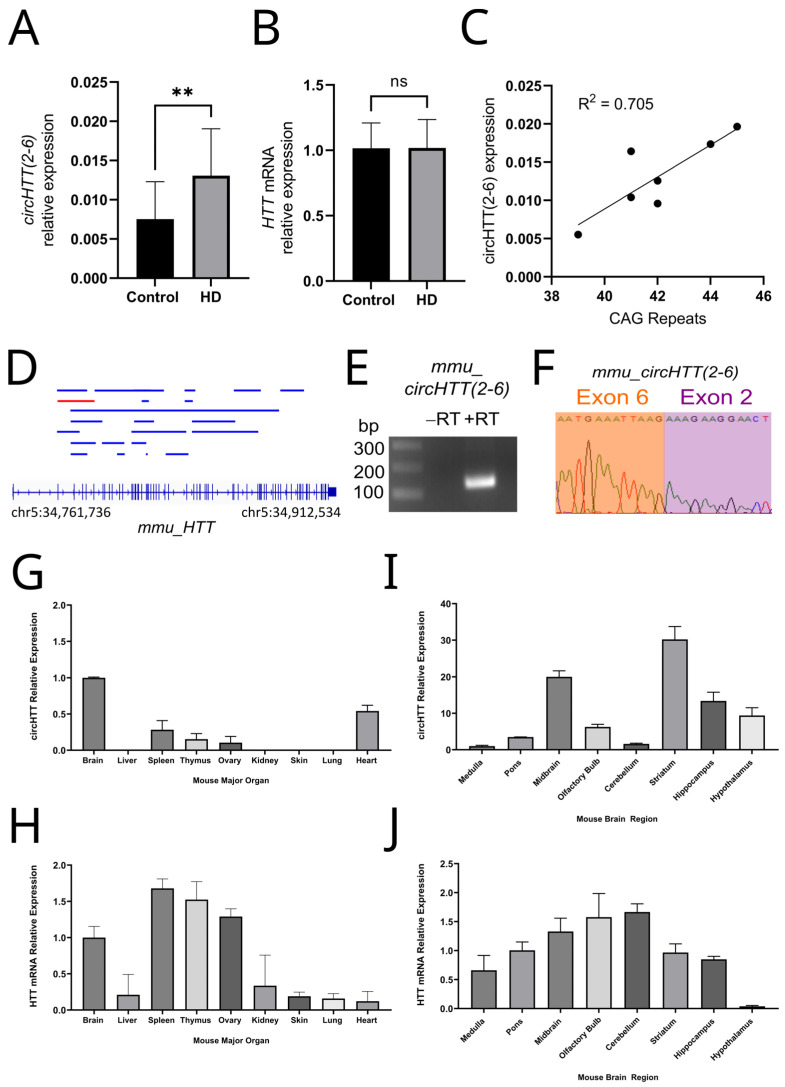
Clinical relevance of *circHTT(2-6)* and brain enrichment of its mouse orthologue, *mmu_ circHTT(2-6)*. QRT-PCR analysis shows a statistically significant difference in (**A**) *circHTT(2-6)* expression, but not (**B**) *HTT* mRNA between control (*n* = 4) and Huntington disease frontal lobes (*n* = 7). ** *p* = 0.01 (Student’s *t*-test, two-tailed); ns: not significant. Data presented as mean ± standard deviation, normalised to *TBP*. (**C**) Positive correlation between *circHTT(2-6)* expression and CAG repeat numbers from 7 HD patients (R^2^ = 0.705 and *p* = 0.01). Statistical analysis performed by Pearson’s correlation. (**D**) All known circRNAs from the mouse *HTT* gene from circBase and CIRCpedia v2. *HTT* gene locus shown at bottom, with vertical blue lines representing exons. Blue lines represent individual circRNAs, with *mmu_circHTT(2-6)* highlighted in red. (**E**) Agarose gel electrophoresis showing *mmu_circHTT(2-6)* amplification from mouse brain RNA. –RT: no RT control. (**F**) Sanger sequencing chromatogram of *mmu_circHTT(2-6)* amplicon showing backsplice junction between exons 6 and 2. QRT-PCR amplification of *mmu_circHTT(2-6)* and *mmu_HTT* mRNA from various (**G**,**H**) major mouse organs and (**I**,**J**) mouse brain regions. QRT-PCR data presented as relative expression of transcripts, mean ± standard deviation, normalised to *GAPDH*.

In order to assess the relevance of *circHTT(2-6)* in the context of HD, we compared its expression in the cortex of brains impacted by HD from the frontal lobe. Harvesting RNA from seven HD and four control patient brains, we performed qRT-PCR in order to quantify *circHTT(2-6)* and the cognate mRNA, *HTT*. This demonstrated that *circHTT(2-6)* was significantly more abundant, 1.9-fold higher, in the frontal lobes of the HD patients compared to those of the controls (Figure 2A) (Student’s *t*-test, *p* = 0.01). This was despite no significant difference in the abundance of *HTT* mRNA between the two cohorts (Figure 2B). Within the HD cohort, we also investigated the correlation between the abundance of *circHTT(2-6)* and the number of CAG repeat regions in *HTT*. Using the largest CAG repeat number allele for each patient, a significant positive correlation was found between CAG repeat numbers and *circHTT(2-6)* expression (Figure 2C, Pearson’s correlation coefficient R^2^ = 0.705; *p* = 0.01).

### 3.3. Expression Profiling of Mouse Orthologue, mmucircHTT(2-6)

In order to examine whether this circRNA was conserved in mice, we searched public circRNA databases. Within the 22 identified circRNAs from the *HTT* gene from CIRCpedia, we identified the mouse ortholog of *circHTT(2-6),* MMU_CIRCpedia_17475, comprising exons 2–6 (Figure 2D, Supplementary Table S2), and this was amplified by divergent RT-PCR from RNA isolated from mouse brain, with the backsplice junction confirmed by Sanger sequencing (Figure 2E,F). The abundances of the *mmu_circHTT(2-6)* and *mmu_HTT* mRNA were assessed by qRT-PCR in nine major mouse organs—brain, liver, spleen, thymus, ovary, kidney, skin, lung, and heart. While the *HTT* mRNA was detected in all tissues, mmu_*circHTT(2-6)* was only found to be present in the brain, heart, spleen, thymus, and ovary, with the brain displaying the highest absolute expression of mmu_*circHTT(2-6)* (Figure 2G,H). To better dissect the expression within the mouse brain, eight regions were microdissected with RNA purified from the medulla, pons, midbrain, olfactory bulb, cerebellum, striatum, hippocampus, and hypothalamus. The highest expression of *mmu_circHTT(2-6)* was found in the striatum, which displayed the fourth-lowest abundance of *mmuHTT* mRNA (Figure 2I,J). The highest conversion efficiency of circRNA, calculated by comparing the *mmu_circHTT(2-6)* abundance as a proportion of the *HTT* mRNA abundance, was in the hypothalamus.

### 3.4. Overexpression of circHTT(2-6)

**Figure 3:**
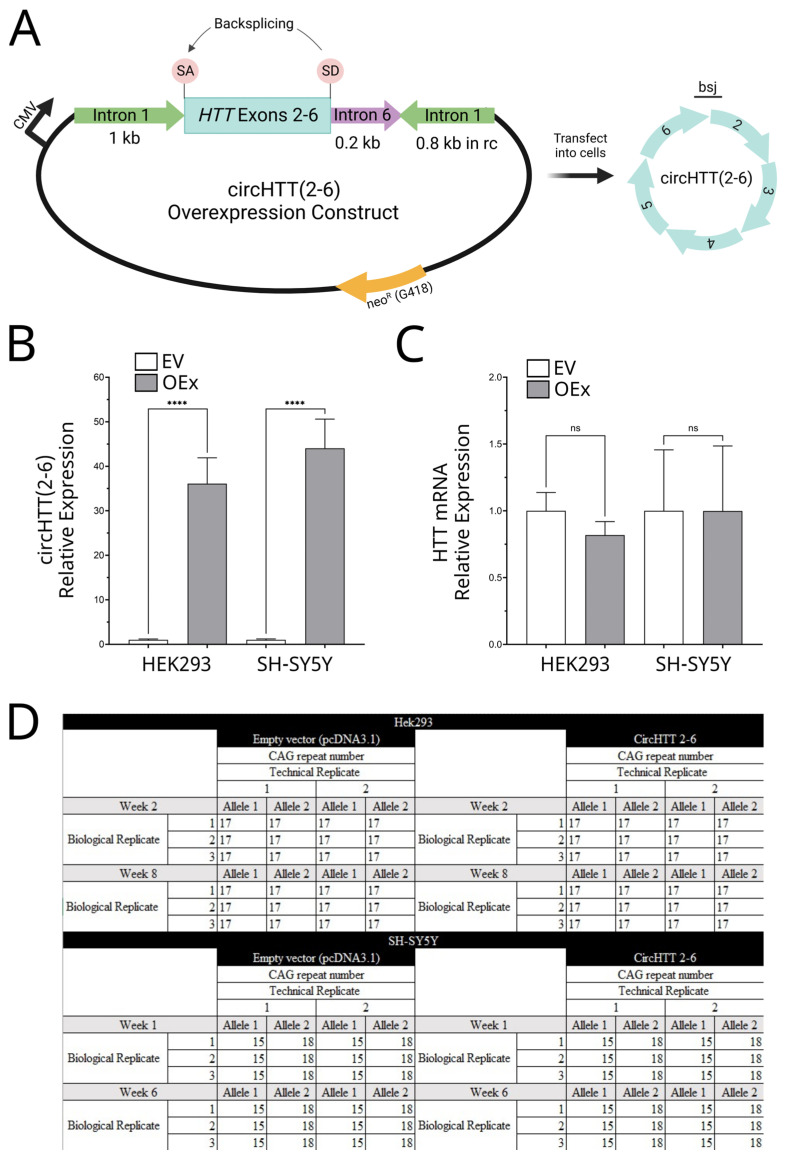
Profiling of transcriptional and CAG repeats following overexpression of *circHTT(2-6)*. (**A**) Vector construct to achieve overexpression of *circHTT(2-6)* using pcDNA3.1 backbone, showing structure of *circHTT(2-6)*. SA: splicing acceptor, SD: splicing donor, rc: reverse complement, bsj: backsplice junction. Created with biorender.com. QRT-PCR to compare expression levels between empty vector (EV) and *circHTT(2-6)* overexpression (OEx) lines in both HEK293 and SH-SY5Y cells at five weeks post-selection (*n* = 3 biological replicates) for (**B**) *circHTT(2-6)* and (**C**) *HTT* mRNA. Statistics performed by Student’s *t*-test, two-tailed; **** *p* < 0.0001; ns: not significant. Data shows mean ± standard deviation, normalised to *GAPDH*. (**D**) CAG repeat lengths for two alleles in HEK293 (top) and SH-SY5Y (bottom) cells comparing empty vector (pcDNA3.1) and *circHTT(2-6)* overexpressing cell lines. *n* = 3 biological replicates for each cell line, with each assay completed with 2 technical replicates.

Given the enrichment of *circHTT(2-6)* in the brains of HD patients and indications that the expression of its mouse orthologue is within the regions most affected by HD pathogenesis, we cloned an overexpression construct for *circHTT(2-6)* in order to investigate the function of this circRNA. This construct was assembled as described [15,23] in the vector pcDNA3.1 (Figure 3A), and three biological replicates of cells, transfected with either the *circHTT(2-6)* construct (OEx) or pcDNA3.1 (EV), were established in two human cell lines: human embryonic kidney (HEK293) and neuroblastoma (SH-SY5Y) cells. Following G418 selection of transformants for seven days (Supplementary Figure S1), qRT-PCR was performed. *CircHTT(2-6)* was found to be overexpressed 72–94-fold compared to empty vector controls across all biological replicates of HEK293 cells and SH-SY5Y (Figure 3B, *p* < 0.0001). This level of expression was found to be consistent for up to eight weeks of culture (Supplementary Figures S2 and S3). Linear *HTT* expression was also investigated (Figure 3C), but this was not found to be significantly affected by *circHTT(2-6)* overexpression in either HEK293 cells or SH-SY5Y cells.

### 3.5. HD Diagnostic PCR

Once *circHTT(2-6)* overexpression was confirmed, the phenotypic and genotypic consequences of *circHTT(2-6)* overexpression could be investigated, such as investigating if *circHTT(2-6)* overexpression has any effect on CAG repeat numbers. Genomic DNA was collected from HEK293 and SH-SY5Y empty vector and *circHTT(2-6)* overexpression cell lines at an early post-selection timepoint (1–2 weeks) and after an extended time in culture (6 weeks for SH-SY5Y and 8 weeks for HEK293 cells). These samples then underwent an HD diagnostic PCR assay to determine if *circHTT(2-6)* overexpression caused changes in the CAG repeat number. HEK293 cells exhibited 17 CAG repeats at both loci, while SH-SY5Y cells had 2 different repeat alleles, at 15 and 18 repeats. Compared to empty vector controls, no difference was seen in CAG repeat number in the *circHTT(2-6)* overexpression lines for either HEK293 or SH-SY5Y cell lines (Figure 3D).

### 3.6. Cell Morphology Analysis

As changes in nuclear and cell morphology have been noted in HD [24,25], these same parameters were also investigated in *circHTT(2-6)* overexpression cell lines. This was achieved through high-content imaging using an Operetta CLS (Perkin Elmer). In combination with specific cellular fluorescent stains, including DAPI (nucleus), Phalloidin (F-actin filaments), and CellTracker Deep Red (cytoplasm), the analysis could illuminate a range of cellular and nuclear dimensions, including size and volume (Supplementary Figure S4).

**Figure 4:**
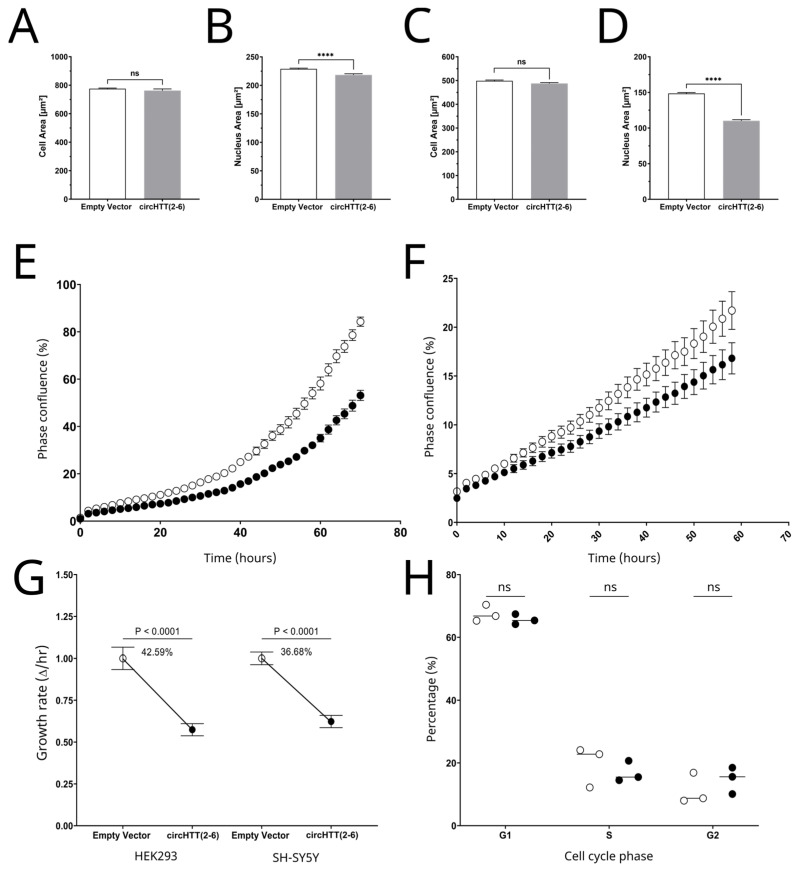
Overexpression of *circHTT(2-6)* in HEK293 and SH-SY5Y cells decrease nuclear area and cell proliferation independent of cell size and cell cycle progression. Cell morphology analysis of HEK293 cells for (**A**) cell area and (**B**) nuclear area and SH-SY5Y cells for (**C**) cell area and (**D**) nuclear area at 5 weeks after selection. Mean ± standard error. Statistical test performed by Student’s *t*-test for *n* = 6000 cells across three biological replicates (alpha decreased to 0.01 due to large number of cells utilised), **** *p* < 0.0001, ns: not significant. Cell proliferation based on cell confluence for one representative biological replicate of EV (unfilled circles) and *circHTT(2-6)* overexpression (filled circles) 5 weeks after selection for (**E**) HEK293 cells and (**F**) SH-SY5Y cells. *n* = 2000 individual cells for each sample. (**G**) Maximum growth rate based on phase object confluence (%) comparing empty vector and *circHTT(2-6)* overexpression 5 weeks after selection for HEK293 and SH-SY5Y cells, respectively. Statistical analysis performed by one-way ANOVA. (**H**) Relative distribution of cell cycle stages (G1, S, G2) of asynchronous empty vector (unfilled circles) and *circHTT(2-6)* overexpression (filled circles) SH-SY5Y cells. *n* = three biological replicates, with the mean represented by a horizontal line. Student’s *t*-test, ns: not significant.

While no difference was seen in the cell size (µm^2^) between the control and *circHTT(2-6)* overexpressing HEK293 cells, a moderate, but statistically significant, 5% decrease was seen for nuclear area (µm^2^) at five weeks after selection (Figure 4A,B, *p* < 0.0001, two-tailed *t*-test). Importantly, the same trends were replicated in SH-SY5Y cells, with a much larger decrease in nuclear area (25%) with no significant change to the cell area (Figure 4C,D).

### 3.7. Cell Proliferation

Cell proliferation was assessed in cells overexpressing *circHTT(2-6)* through Incucyte high-content imaging. We employed a strategy to measure proliferation based on cell confluence, which was informed by our earlier observation that the circRNA did not elicit a change in cell size. These analyses demonstrated that overexpression of *circHTT(2-6)* reduces cell proliferation in both HEK293 (Figure 4E) and SH-SY5Y cells (Figure 4F). We compared the maximum growth rate of each cell line over a 10 h exponential period for all three replicates, which showed a statistically significant decrease from *circHTT(2-6)* overexpression in HEK293 (17.41%, *p* = 0.0002, two-way ANOVA) and SH-SY5Y cells (37.68%, *p* = 0.0001) (Figure 4G). In order to address whether this result was a non-specific response to overexpression of a circRNA, we overexpressed another unrelated circRNA, *circNFASC(26-27)*, in HEK293 cells. In contrast to *circHTT(2-6)*, overexpression of *circNFASC(26-27)* resulted in no significant change in cell proliferation and maximum growth rate (Supplementary Figure S5).

### 3.8. Cell Cycle Analysis by Flow Cytometry

Given the decreased rate of cell proliferation, it was hypothesised that there may be some alteration in the rate of cell cycle progression as a result of the overexpression of *circHTT(2-6)*. This was investigated by flow cytometry cell cycle analysis using Hoechst 34,580 live cell DNA dye to measure the DNA content per cell. Three biological replicates of asynchronous SH-SY5Y cells of empty vector and *circHTT(2-6)* overexpression were collected at a phase confluency of approximately 50%, stained with Hoechst dye, and then underwent quantitative flow cytometry.

After doublets were removed, it was found that there was no statistically significant difference between the empty vector and *circHTT(2-6)* overexpressing cell lines for the proportion of cells present in each phase of the cell cycle (Figure 4H, *p* = 0.6206, 0.4300 and 0.3244 for G1, S and G2 phase, respectively). These data suggest that the observed reduction in proliferation is accompanied by a proportional reduction in the length of each phase of the cell cycle, rather than one specific phase or phase transition being affected.

### 3.9. HTT Protein Quantification and Subcellular Localisation

**Figure 5:**
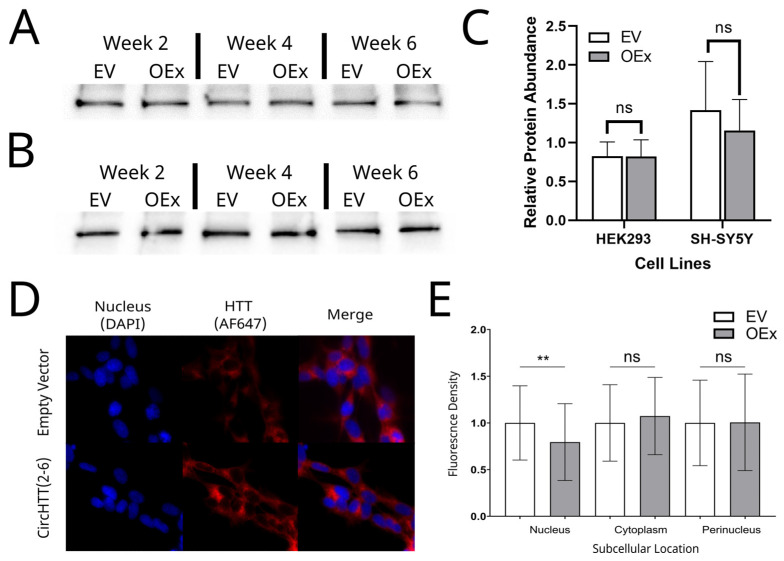
Nuclear HTT protein localisation, but not total HTT protein, is reduced with *circHTT(2-6)* overexpression. Western blot using α-HTT antibody (Abcam, ab109115) and goat anti-rabbit-HRP secondary at 2, 4, and 6 weeks post-selection for empty vector (EV) and *circHTT(2-6)* overexpression (OEx) lines of (**A**) HEK293 and (**B**) SH-SY5Y cells. TPC: total protein staining value shown for each lane. (**C**) Relative HTT abundance calculated from western blot images, showing average HTT protein abundance + standard deviation (*n* = 3 timepoints). (**D**) Representative immunofluorescence images of empty vector and *circHTT(2-6)* overexpressing SH-SY5Y cells. Nuclear (DAPI) shown in blue, AlexaFluor 647 (HTT localisation) shown in red along with merged image. (**E**) Density of pixels within region of interest (nucleus, cytoplasm, and perinucleus) in three biological replicates of empty vector (*n* = 88 cells) and *circHTT(2-6)* overexpression (*n* = 80 cells) SH-SY5Y cells. Data points show the mean and standard deviation of all three biological replicates for fluorescence signal density within a region of interest Statistical analysis performed by one-way ANOVA, ** *p* < 0.01, ns: not significant.

With such little known about the HTT protein, we next sought to evaluate the effect of *circHTT(2-6)* overexpression on its function. First, we investigated whether the circRNA influenced HTT protein abundance. This was accomplished by Western blotting of the HTT protein in HEK293 cells (Figure 5A) and SH-SY5Y (Figure 5B) cells at three timepoints (2, 4, and 6 weeks post-selection). Normalising to total protein load, the abundance of HTT protein was not significantly altered by *circHTT(2-6)* overexpression in either cell line (Figure 5C), suggesting that *circHTT(2-6)* overexpression has no effect on HTT protein abundance.

We then sought to assess the subcellular localisation of the HTT protein, which is known to be affected in HD patients, although mostly linked to the mutant HTT, rather than wild-type HTT [26]. To visualise wild-type HTT protein localisation, immunofluorescence was utilised with the same antibody used for western blot. Representative immunofluorescence images for SH-SY5Y cells are shown in Figure 5D, where HTT protein is both nuclear and cytoplasmic in EV and *circHTT(2-6)* overexpression lines. In order to achieve quantitative analysis of HTT protein localisation within different cellular compartments, nucleus, perinucleus, and cytoplasm, ImageJ was utilized (region-specific masks are shown in Supplementary Figure S6). Quantitative analysis revealed that there was a decrease of ~24% in HTT protein abundance in the nucleus of *circHTT(2-6)*-overexpressing SH-SY5Y cells (Figure 5E, *p*= 0.0069). While there was a 5% increase in the levels of HTT in the cytoplasm, there was no statistically significant change in the abundance in the cytoplasm and perinucleus (*p* = 0.6185 and 0.9995 respectively).

## 4. Discussion

Research within the last decade has demonstrated that circRNAs are not only evolutionarily conserved, but also demonstrate differential expression between cells, tissues, and diseases, thus suggesting that circRNAs may have functional significance. The exact functions of these circRNAs, however, let alone the consequences of differential expression in regard to disease pathology, remain elusive [27]. CircRNAs, while present in all tissues, are most abundant in neurological tissue [28] and vary in expression depending on the location within the brain [29]. Therefore, it is not surprising that differential expressions of circRNAs have been linked to a range of neurological diseases [16]. However, such characterisation of circRNAs has yet to be extended to rarer neurological diseases, such as Huntington’s disease (HD).

This study attempted to close this gap by investigating the genotypic and phenotypic consequences of overexpressing the first identified circRNA from the *Huntingtin* gene (*HTT*), containing exons 2, 3, 4, 5, and 6 *circHTT(2-6)*. Clinical relevance for this circRNA was substantiated by finding a 1.9-fold increase in the abundance of *circHTT(2-6)* within the cerebral cortex of the posterior frontal lobes of HD patient brains, compared with those of healthy controls. The posterior frontal lobe was chosen due to its well-established role in dysfunctional fronto-striatal connectivity, as previously discussed, which is heavily impacted in HD patients [30]. Of course, given that we only had access to brain tissue from seven HD patients and four healthy controls, we acknowledge that a larger sample size would allow for a more powerful analysis of this association.

The mouse orthologue, mmu_*circHTT(2-6)*, was not only enriched in the brain, but also within the striatum. The main projecting neurons within the striatum are medium spiny neurons (MSNs), which are the cell type in the brain which is significantly reduced in HD pathology [31]. It was, therefore, encouraging to observe an effect from manipulating the expression of this circRNA in two cell lines, HEK293 and SH-SY5Y. SH-SY5Y cells comprise a neuroblastoma cell line and are commonly utilised in HD research [21,22]. Reduced cell proliferation and altered nuclear morphology have been noted in HD post mortem brains previously [25]. Furthermore, in HD patients, mutant and wild-type HTT proteins are found to be nuclear-localised, which is largely thought to be due to the propensity of mutant HTT to be highly promiscuous and bind various proteins, including the nuclear master regulator REST within the cytoplasm [32]. Here, we found wild-type HTT to be less abundant in the nucleus; therefore, it would be interesting to determine whether *circHTT(2-6)* may interact with the HTT protein, or impact nuclear protein import/export.

As we previously found *circHTT(2-6)* to be present in the nucleus and cytoplasm of human cells [15], we considered that there may be impacts on cell transcription, or even on the CAG repeat region itself. Indeed, the abundance of *circHTT(2-6)* in HD brains was very strongly associated with CAG repeat numbers (R^2^ = 0.705; Figure 2C). However, it was found that neither *HTT* mRNA nor the CAG repeat numbers were affected within the eight-week study window for either cell line following *circHTT(2-6)* overexpression. While we concluded that *circHTT(2-6)* does not influence CAG repeat numbers, this experiment is limited, as increases in CAG repeat number occur over years [33,34] and cannot be faithfully recapitulated in cell culture. Alternatively, even if *circHTT(2-6)* did not contribute to the CAG expansion number, it cannot be ruled out that it has no effect on disease pathogenesis at all. It is possible, for example, that *circHTT(2-6)* overexpression may be a consequence of an increased CAG repeat number, to which *circHTT(2-6)* may act on other areas of the cell to induce disease phenotypes. Probing the EMBL-EBI gene expression mouse atlas, we identified the mouse orthologues of circRNA biogenesis proteins—Nova1, QK1, FUS, MBNL1, and ADAR1—were expressed in each of the tissues and brain regions where *mmu_circHTT(2-6)* was found. Three factors—NOVA1, QK1, and ADAR1—each showed significant positive correlations with *mmu_circHTT(2-6)* expression in these tissues, with *NOVA1* showing the strongest correlation across all tissues (Pearson’s correlation R^2^ = 0.904; *p* = 0.0003) (Supplementary Figure S7). Liu et al. demonstrated interaction of NOVA1 with circUVRAG using the RNA-Protein Interaction Prediction (RPISeq) program (http://pridb.gdcb.iastate.edu/RPISeq/, accessed on 2 March 2023) [35]. RPIseq calculated a 91% probability of mouse NOVA1 interacting with *mmu_circHTT(2-6)* (accessed 26 March 2023). Further support for the role of NOVA1 in biogenesis of *mmu_circHTT(2-6)* is that the hypothalamus, which is also affected in HD, displays the greatest *mmu_circHTT(2-6)* conversion efficiency from *HTT* mRNA, and the hypothalamus has been shown to be enriched for and functionally dependent on NOVA1 expression [36]. While beyond the scope of this report, it would be worth further investigation into NOVA1 and other factor(s) underlying *mmu_circHTT(2-6)* biogenesis.

Another phenotypic consequence of *circHTT(2-6)* overexpression we observed was reduced cell proliferation. This process is tightly controlled across all cell types, and alterations of a range of biochemical processes involved with cell proliferation regulation have been associated with several neurodegenerative disorders [37,38,39]. *CircHTT(2-6)* overexpression led to a decrease in cell proliferation in both HEK293 and SH-SY5Y cells. One report found a similar decrease in proliferation, with unaffected cell cycle distribution, in the skin fibroblasts of eight HD patients and seven matched healthy controls [34]. The authors found this was associated with altered mitochondrial activity in HD fibroblasts. On the contrary, another report observed a significant increase in cell proliferation in the subependymal layer (SEL) of HD brain tissue [40]. This study included nine HD brains and six control brains, and cell proliferation analysis was measured by immunofluorescence of brain tissue for proliferating cell nuclear antigen (PCNA), which exclusively labels cells within the S phase of the cell cycle. The results showed an increased number of PCNA cells within the SEL of HD brains compared to the control brains. The results also showed a statistically significant correlation between the number of PCNA cells, HD neuropathological grade (*p* < 0.003) and number of CAG repeats within the expanded allele of the HD gene (*p* < 0.002). The authors suggest that cell proliferation increases as a response to neuronal cell loss within the caudate nucleus, which is the section of the brain found to be severely affected in HD. The results from these papers as well as our own suggest that altered cell proliferation occurs differently during the early and later stages of disease development.

In order to investigate cell proliferation further, we investigated the consequences of *circHTT(2-6)* overexpression on nuclear and cellular morphology. Nuclear architecture has been researched in various diseases; however, this is limited in neurological disease [41]. A report by de Castro et al. investigated changes in HD nuclear morphology in HD blood cells [24]. Results showed that the nuclear area in peripheral blood mononuclear cells of early and moderate HD patients increased compared to control patients (6% and 8.8% for early and moderate HD patients respectively, *p* <0.001 for both groups). Our study, however, showed the opposite with *circHTT(2-6)* overexpression in HEK293 and SH-SY5Y lines, which saw a decrease in nuclear area. Whilst analysis of HD blood offers possible benefits, such as the identification of biomarkers [42] and indicators of disease progression, it is not the blood that is predominantly affected in HD, but rather the basal ganglia and the medium spiny neurons that are the most affected. Therefore, changes in nuclear area and circRNA expression within the blood may not correlate with disease pathogenesis occurring within the brain. Another report investigated the percentage of abnormal nuclei within the cortex and striatum of wild-type and HD mouse models [43]. Indeed, the percentage of abnormal nuclei was found to increase from 21% to 39% within the cortex of wild-type mice, which were homozygous for seven polyglutamine repeats (HTT Q7/7). In mouse models heterozygous and homozygous for the polyglutamine repeat (HTT Q7/175 and HTT Q 175/175), the percentages of abnormal nuclei within the cortex increased to 72% and 89% in heterozygous and homozygous mice, respectively. The authors extended their findings to humans by investigating nuclear morphology in iPSC-derived neuronal progenitors from HD and control individuals. Indeed, 55% of nuclei had misshaped nuclear envelopes compared to 16% seen in control cells. The significant differences in nuclear area found in our study may thus be an indication of early HD pathogenesis.

Precisely how the mHTT protein contributes to HD pathology remains unclear; however, it is necessary for HD pathogenesis [44]. Research has indicated that mutated HTT protein may influence a range of cellular processes such as transcription, axonal transport, cytoskeleton structure and function, and autophagy [45]. The effects of *circHTT(2-6)* overexpression on HTT protein abundance and localisation demonstrated that, while no consistent change in protein abundance was seen, it did alter the subcellular distribution, with a decrease in nuclear localisation. Studies have demonstrated that the mHTT protein is localised to the nucleus, whilst the wild-type HTT protein is more cytoplasmic [46]. Another study investigated HTT localisation between non-neural and neuronal cells and found that HTT localisation was predominantly cytoplasmic in non-neuronal cells, whilst was more nuclear in neural cells [47]. The ability of mHTT to promote nuclear localisation of the RE1-silencing transcription factor (REST) and suppression of brain-derived neurotrophic factor (BDNF) expression is believed to be linked to the progressive death of neurons [48,49]. This is particularly interesting when compared with our results, where HTT becomes less nuclear with *circHTT(2-6)* overexpression in SH-SY5Y cells, which represent a neuronal cell line. While beyond the scope of this study, it would be interesting to assess the capacity of *circHTT(2-6)* to alter the localisation of mutant HTT, perhaps using HD iPSCs, which may have a role in ameliorating the severity of HD.

It has been reported that the nuclear pore complex (NPC), which is responsible for transporting the HTT protein between the nucleus and cytoplasm, is dysregulated in HD [50]. To that end, active transport through the NPC is achieved through GTPase RAN and RANGP1. NPC dysfunction has indeed been reported in HD, due to the mislocalisation of both RAN and RANGAP1 uniquely in medium spiny neurons derived from HD iPSCs varying in CAG repeat length [50,51]. Interestingly Lamin B, which plays pivotal roles in RNA nuclear export and NPC organisation, both of which are altered in HD, are significantly higher in the striatum of HD brains compared to those of healthy controls [26,52]. This is particularly interesting, as *circHTT(2-6)* was also found to be overly expressed within the frontal cortex of HD brains.

HD is a fatal neurodegenerative disease for which there is currently no cure. An understanding of the molecular mechanisms responsible for HD is essential to find curative treatment. However, the cause of the CAG repeat mutation responsible for HD remains unknown, and the function of the HTT protein remains highly debated within the literature. Here, we show that the circRNA containing exons 2, 3, 4, 5, and 6 of the *HTT* gene (*circHTT(2-6)*) is more abundant within human HD brains, and the mouse orthologue is enriched in mouse brains. Overexpression of *circHTT(2-6)* in HEK293 and SH-SY5Y cells demonstrated no change to the CAG repeat region responsible for HD, but resulted in a decrease in cell proliferation, nuclear area, and altered subcellular localisation of the HTT protein. These results were in agreeance with known pathophysiological changes in HD, suggesting that further research should be undertaken to investigate the functional mechanisms of *circHTT(2-6),* particularly in vivo, and its role in the mislocalisation of HTT in contributing to HD pathogenesis.

## Figures and Tables

**Table 1:** Huntington’s disease (HD) and healthy control patient samples used in this study.

Sample ID	Sex	Age at Death	Clinical Group (HD or Control)
SA0080	Female	81	HD
SA0091	Male	71	HD
SA0110	Male	72	HD
SA0167	Female	63	HD
SA0188	Male	64	HD
SA0263	Male	65	HD
SA0272	Male	60	HD
SA0096	Female	73	Control
SA0162	Male	72	Control
SA0214	Male	64	Control
SA0230	Male	86	Control

## Data Availability

Not applicable.

## Supplementary Materials

The following are available online at www.mdpi.com/article/10.3390/cells12091337/s1, Table S1: Oligonucleotides used in this study. Table S2: CircRNAs from HTT in humans and mice from CIRCpedia. Figure S1: Selection of HEK293 cells after seven days of exposure to G418. Figure S2: *circHTT(2-6)* expression in three biological replicates of empty vector (pcDNA3.1) and *circHTT(2-6)* overexpressor HEK293 cells. Figure S3: *circHTT(2-6)* expression in three biological replicates of empty vector (pcDNA3.1) and *circHTT(2-6)* overexpressor SH-SY5Y cells. Figure S4: DAPI, Phalloidin and Cell Tracker Red dyes used for operetta analysis. Figure S5: Cell proliferation of *circNFASC(26-27)* in HEK293 cells. Figure S6: Nuclear, Cytoplasmic and Perinuclear boundaries used to measure HTT immunofluorescence. Figure S7: Correlation of expression of known/orthologous circRNA biogenesis factors with *mmu_circHTT(2-6)* in mouse tissue.
