# Supplementary material for: Functional Characterisation of the Circular RNA, circHTT(2-6), in Huntington’s Disease
Source: Cells. 2023 May 7;12(9):1337. doi: 10.3390/cells12091337 (PMC10177161; doi:10.3390/cells12091337)
Supplement: Supplementary file 1 [file cells-12-01337-s001.zip › Cells GANTLEY Supp Files.pdf]

cells

Supplementary Table S1: *Oligonucleotides used in this study.*

Target	***Oligonucleotide sequence (5’-3’)***
Human circHTT(2-6) qRT-PCR	*F (exon 6): GAGACCCGAAGAATCAGTCCA*<br>*R (exon 2): CACGGTCTTTCTTGGTAGCTG*
Human HTT mRNA qRT-PCR	*F (exon 64): TTGCTATGGAGCGGGTATC*<br>*R (exon 65): GTATGGCTGCTGGTTGGAC*
Human/ mouse GAPDH qRT-PCR	*F: CAGTGAGCTTCCCGTTCAG*<br>*R: ACCCAGAAGACTGTGGATGG*
Human TBP qRT-PCR	*F: CACGCCAGCTTCGGAGAGTT*<br>*R: ATCAGTGCCGTGGTTCGTGG*
Mouse mmu_circHTT(2-6) qRT-PCR	*F (exon 6): CGGAGGAATCAGTTCAGGAG*<br>*R (exon 2): TCACACGGTCTTTCTTGGTG*
Mouse mmu_HTT mRNA qRT-PCR	*F (exon 66): CTGTCCCTGTCCAACTTCAC*<br>*R (exon 67): TTCCTCCTCTATCTGGTGTCTG*
HD (CAG)n direct test reporting primers	*HD1 (F): 6’FAM-ATGAAGGCCTTCGAGTCCCTCAAGTCCTTC*<br>*447X (R): GGCGGCGGTGGCGGCTGTTG*

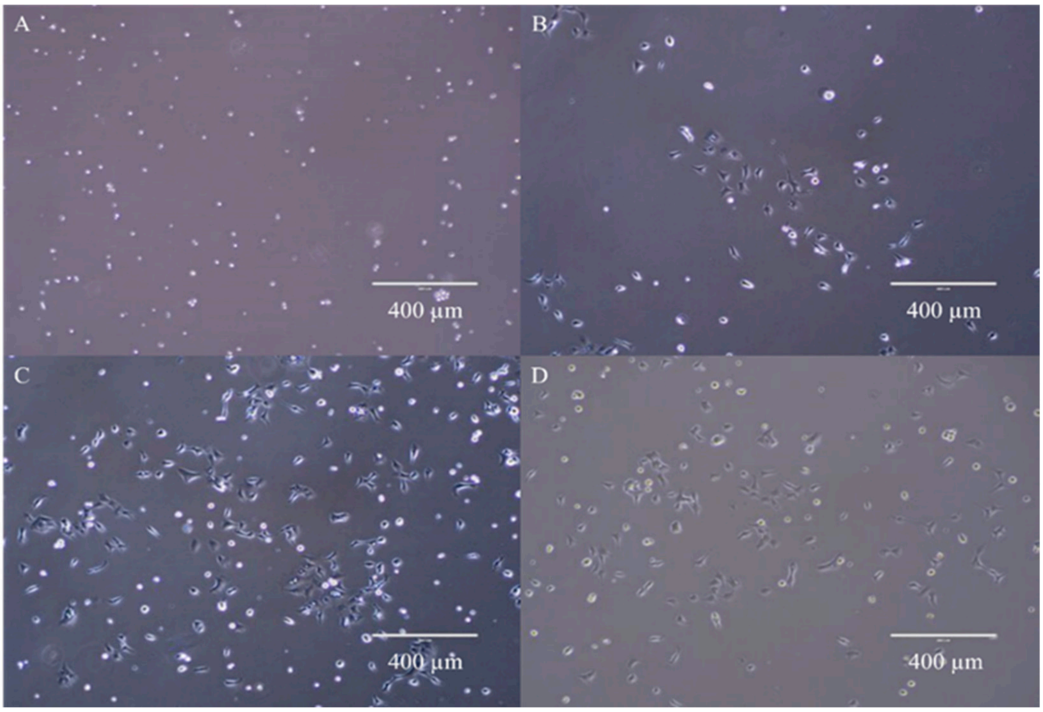

Supplementary Figure S1: *Figure shows transfected HEK293 cells 1 week after G418 selection. (**A**) Non-transfected HEK293 cells killed after seven days of selection, with HEK293 cells surviving following transfection with (**B**) circHTT(2-6) (**C**) circNFASC(26-27) and (**D**) empty vector (pcDNA3.1) . Magnification 10 X, scale bar = 400 µM.*

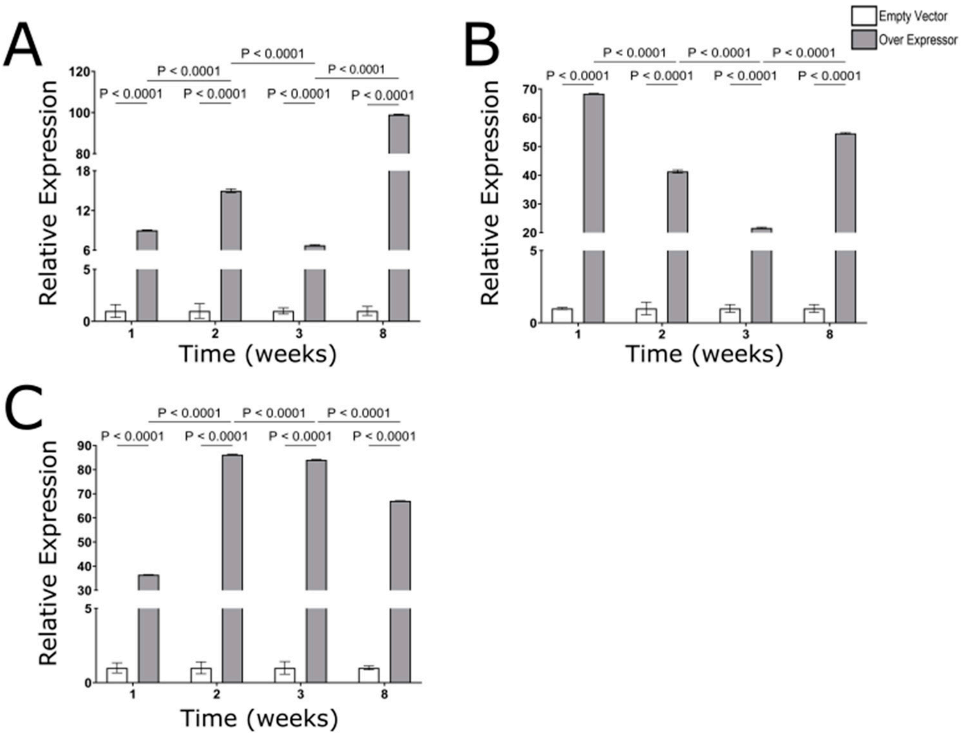

Supplementary Figure S2: *circHTT(2-6) is significantly over expressed in HEK293 cells over eight weeks in 3 biological replicates (one-way ANOVA). Paired empty vector (pcDNA3.1) and circHTT(2-6) overexpression at 1, 2, 3 and 8 weeks after selection of HEK293 cells for* ***A)*** *biological replicate 1,* ***B)*** *biological replicate 2 and* ***C)*** *biological replicate 3. Data shows mean circHTT(2-6) expression and standard deviation normalised to EV, week 2.*

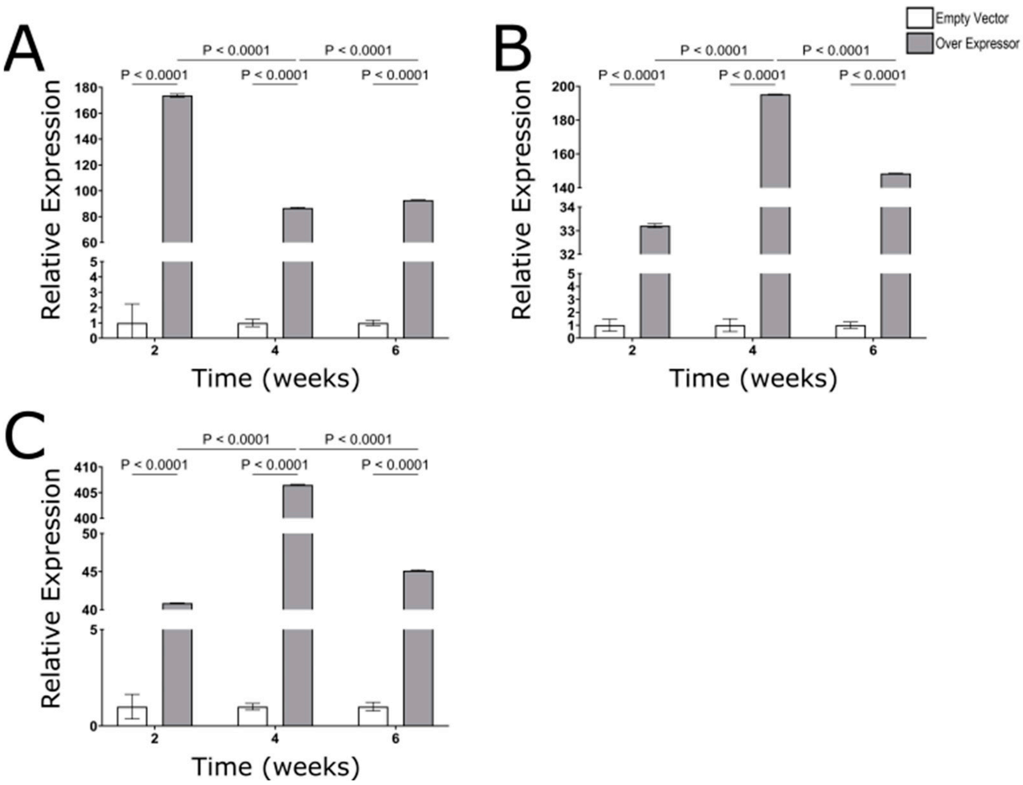

Supplementary Figure S3: *circHTT(2-6) is significantly over expressed in SH-SY5Y cells over six weeks in 3 biological replicates (two-way ANOVA). Paired empty vector (pcDNA3.1) and circHTT(2-6) overexpression at 2, 4 and 6 weeks after selection of SH-SY5Y cells for* ***A)*** *Biological replicate 1,* ***B)*** *Biological replicate 2 and* ***C)*** *Biological replicate 3. Data shows mean circHTT(2-6) expression and standard deviation normalised to EV, week 2.*

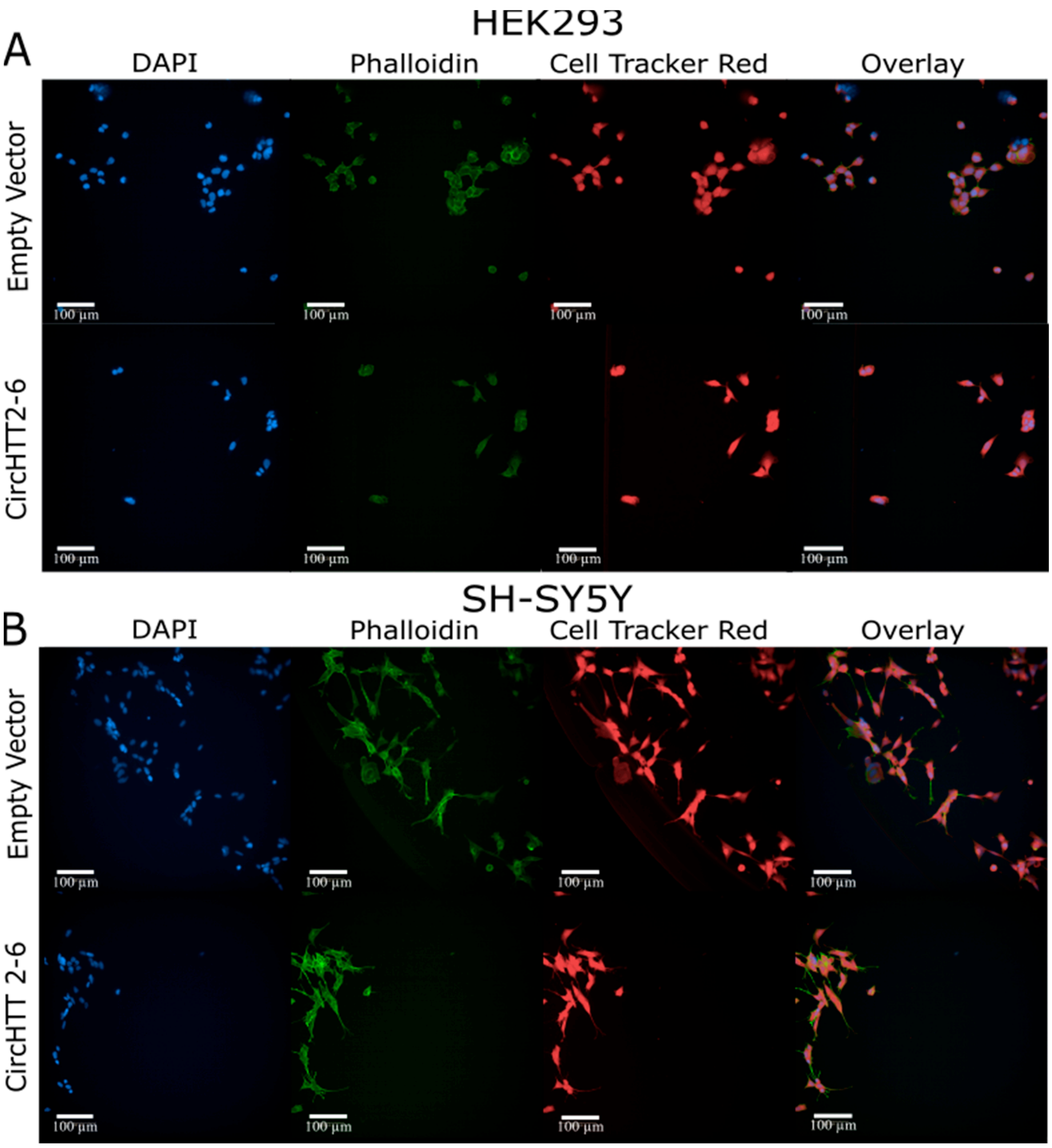

Supplementary Figure S4: *Operetta images of representative biological replicate of* ***(A)*** *HEK293 and* ***(B)*** *SH-SY5Y cells empty vector (pcDNA3.1) and circHTT(2-6) overexpression lines four weeks after selection, stained with DAPI (blue), CellTracker Deep Red (red), Phalloidin (green). 40X magnification, scale bar = 100 µm.*

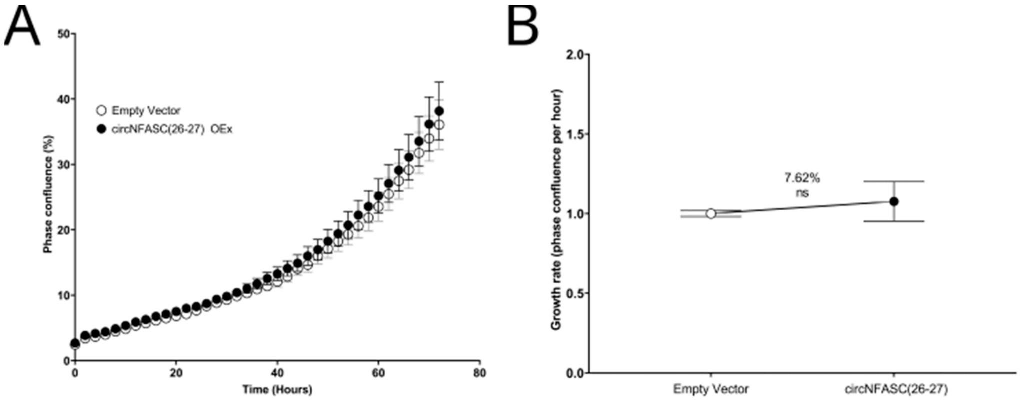

Supplementary Figure S5: *Overexpression of circNFASC(26-27) in HEK293 cells does not affect cell proliferation. (**A**) One biological replicate of empty vector (pcDNA3.1, unfilled circles) and circNFASC(26-27) overexpression lines (filled circles) two weeks after selection of HEK293 cells. Data shows line graph over 72 hours for n = 2000 individual cells from each sample. (**B**) Overall phase object confluence (%) at exponential growth phase (exponential grow phase 10-hour window of linear growth preceding plateauing growth rate between 50-60hrs) for EV and circNFASC(26-27) OEx, showing unaffected cell proliferation rate. ns = not significant. Statistical analysis performed by one-way ANOVA.*

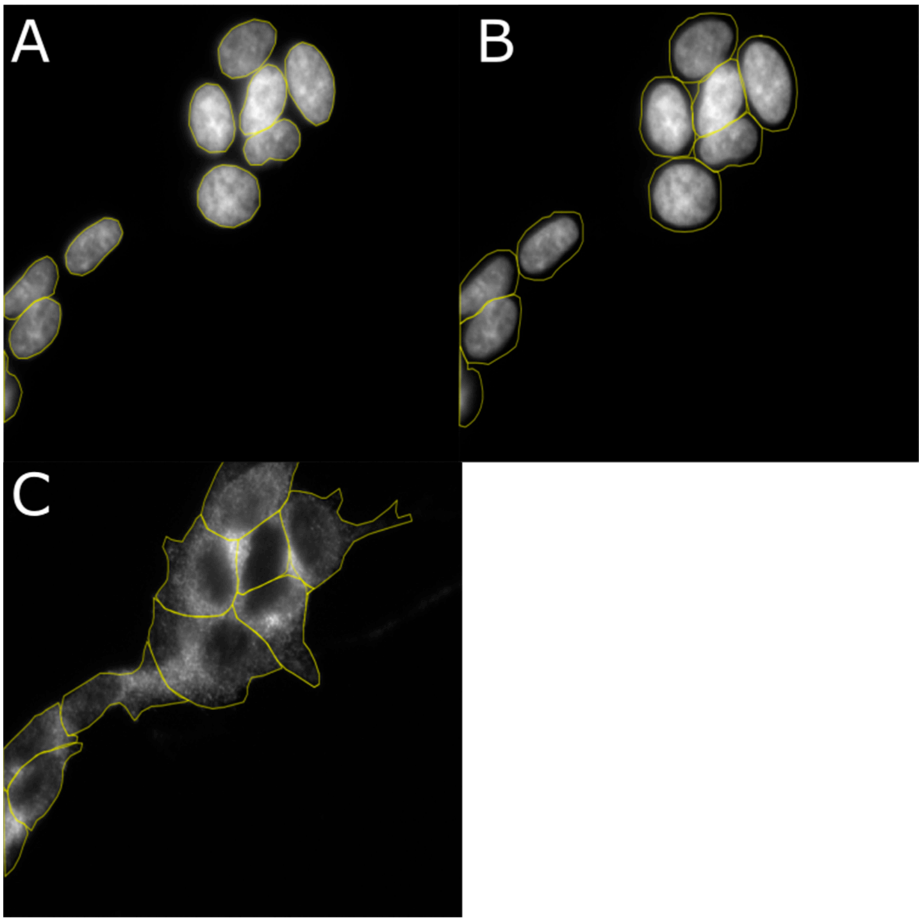

Supplementary Figure S6: *Subcellular boundaries used to measure HTT immunofluorescence. Figure shows one biological replicate of empty vector SH-SY5Y cells six weeks after selection. (**A**) Nuclear boundary, (**B**) perinuclear boundary and (**C**) whole cell boundary (cytoplasm was calculated from this by subtracting the nuclear signal).*

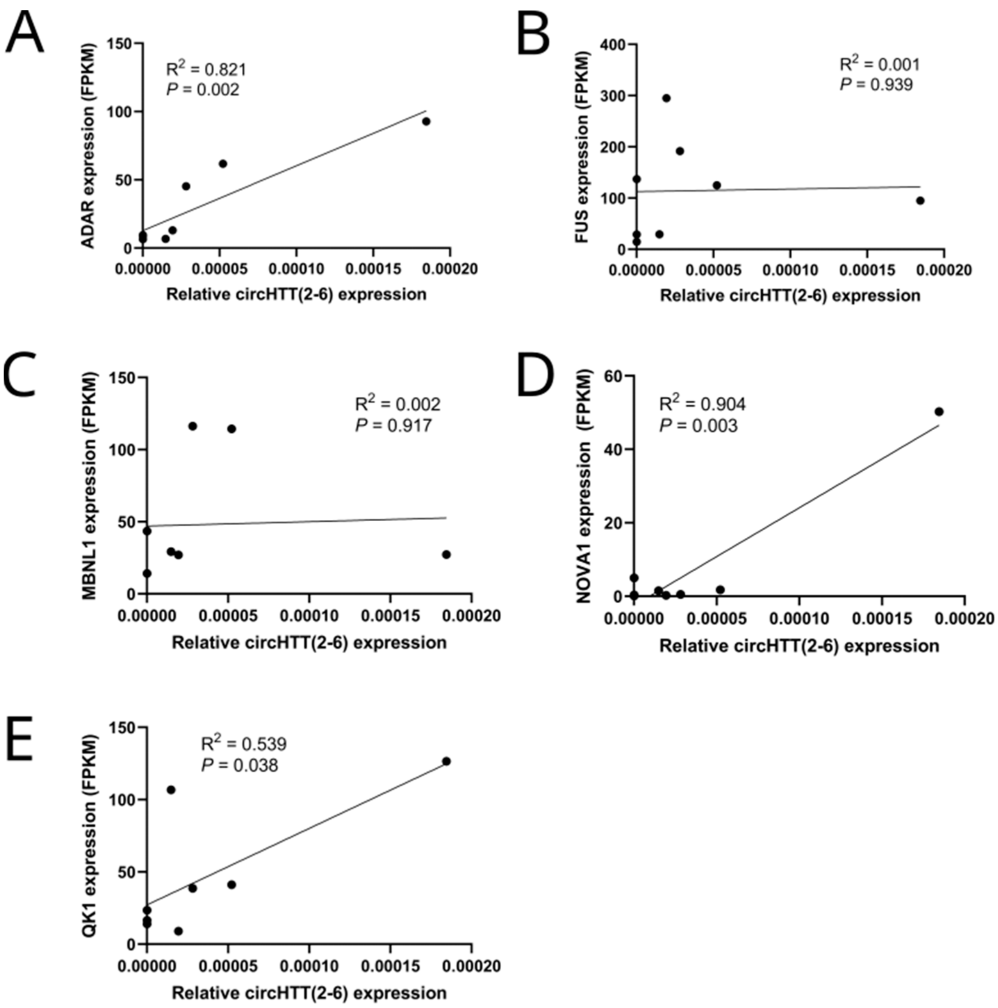

Supplementary Figure S7: *Correlation between expression of mmu_circHTT(2-6) across mouse tissues with known circRNA biogenesis factors. QRT-PCR of mmu_circHTT(2-6) from this manuscript was correlated with RNAseq expression (FPKM) for known circRNA biogenesis factors in mice, or their orthologues (**A**) ADAR (**B**) FUS (**C**) MBNL1 (**D**) NOVA1 (**E**) QK1 with linear regression line fitted and Pearson's correlation coefficient shown as R^2 (and P-value).*
